# Adenocarcinoma with BAC Features Presented as the Nonsolid Nodule Is Prone to Be False-Negative on ^**18**^F-FDG PET/CT

**DOI:** 10.1155/2015/243681

**Published:** 2015-03-24

**Authors:** Hu-bing Wu, Lijuan Wang, Quan-shi Wang, Yan-jian Han, Hong-sheng Li, Wen-lan Zhou, Ying Tian

**Affiliations:** NanFang PET/CT Center, Nanfang Hospital, Southern Medical University, 1838 Guangzhou Avenue North, Guangzhou, Guangdong 510515, China

## Abstract

*Purpose*. The present study investigated which type of adenocarcinoma with BAC features was prone to be false-negative on 18F-FDG PET/CT.* Materials and Methods*. A retrospective study was performed on 51 consecutive patients with localized adenocarcinoma with BAC features. CT and PET were assessed for lesion size, GGO percentage, and SUVmax. Lesions with FDG uptake the same as or more than mediastinal blood-pool activity were considered as PET-positive.* Results*. Of the 51 cases, 19.6% presented as pure GGO nodules, 31.4% as mixed nodules, and 49.0% as solid nodules. None of the pure GGO nodules was 18F-FDG avid, compared with 37.5% of mixed nodules and 96.0% of solid nodules (*χ* ^2^ = 31.55, *P* = 0.000). In the mixed nodule group, SUVmax was negatively correlated with GGO percentage (*r* = −0.588; *P* = 0.021). The positive detection rate of 18F-FDG PET/CT was 50.0%, 55.6%, and 100% in tumors 1.1–2.0 cm, 2.1–3.0 cm, and >3.0 cm in diameter, respectively (*χ* ^2^ = 5.815, *P* = 0.055). General linear model factor analysis showed that the GGO was an important factor contributing to false-negative PET/CT results (*F* = 23.992, *P* = 0.000), but lesion size was not (*F* = 0.602, *P* = 0.866).* Conclusions*. The present study indicated that the adenocarcinoma with BAC features presented as nonsolid nodule is prone to be false negative on 18F-FDG PET/CT.

## 1. Introduction

Bronchioloalveolar carcinoma (BAC) is a subtype of adenocarcinoma that manifests as the lepidic growth of tumor cells along the alveoli without stromal invasion. Whereas, the adenocarcinoma with BAC features is a subtype of adenocarcinoma which comprises a heterogeneous group of tumors with BAC histology mixed with a varying population of invasive cells. Since the latest revision of the WHO/International Association for the Study of Lung Cancer Classification in 2004, the adenocarcinoma with aerogenous spread is referred to as adenocarcinoma with BAC features instead of pure BAC [1, 2]. Lung adenocarcinoma with BAC features is a form of adenocarcinoma with unique clinical, radiological, and epidemiological features. Most of them are noninvasive or minimally invasive carcinoma and associated with a markedly better prognosis compared with invasive adenocarcinoma and may be cured with surgical resection [3, 4].

Adenocarcinoma with BAC features has 3 subtypes: nonmucinous (most frequent), mucinous (25%), and mixed (exceedingly rare). Lee et al. [5] reported that all nonmucinous adenocarcinomas with BAC features appeared as pure ground-glass opacity (GGO) nodules, whereas mucinous ones could appear as solid or part-solid nodules (mixed nodule).

Positron emission tomography (PET)/CT with ^18^F-fluorodeoxyglucose (FDG) is a noninvasive diagnostic technique that provides information about glucose metabolism in lesions and is used routinely for the preoperative staging of NSCLC because of its higher sensitivity and specificity than other diagnostic modalities [6, 7]. However, it has been reported that adenocarcinoma with BAC features has a lower ^18^F-FDG uptake than other types of NSCLC, and is prone to be falsely negative on PET/CT [7, 8]. Nonetheless, it is clear that false-negative does not occur in all the lesions [9, 10]. Therefore, it is needed to determine which type of adenocarcinoma with BAC features is prone to be false-negative and which patient will benefit from postoperative ^18^F-FDG PET/CT surveillance. In the present study, we performed a retrospective study on 51 consecutive patients to investigate what causes the false negativity of adenocarcinoma with BAC features on ^18^F-FDG PET/CT.

## 2. Materials and Methods

### 2.1. Patients

This study was approved by the Institutional Review Board of our hospital. Because of the retrospective nature of the study, the requirement of subject informed consent was waived.

We retrospectively reviewed the records of 51 consecutive patients with adenocarcinoma with BAC features who underwent preoperative PET/CT from December 2005 to January 2011. There were 31 males and 20 females with a mean age of 59 years (range, 35–87 years). Each patient had a focal nodule larger than 1.0 cm. Patients with multiple nodules were excluded. After PET/CT examination, all patients underwent surgical resection within 1 month and a final diagnosis of adenocarcinoma with BAC features was made by histological examination of the surgical specimen. All patients were stage pT1N0 or pT2N0. None of the patients had received prior anticancer treatment before the PET/CT scan, and no patients had insulin dependent diabetes.

### 2.2. ^18^F-FDG PET/CT Examination

PET/CT examinations were performed using a GE Discovery LS PET/CT scanner (GE Healthcare, Waukesha, WI). Patients were instructed to fast for at least 6 hours before the scan, and blood glucose level was monitored by finger stick immediately prior to the study to ensure that their glucose level was within normal levels (<7 mmol/L). Approximately 60 minutes after an intravenous injection of 277 to 444 MBq (7.50–12.00 mCi, 0.15 mCi/kg) of ^18^F-FDG, whole-body PET/CT was performed according to the guidelines for tumor imaging with ^18^F-FDG PET/CT 1.0 [11]. A spiral CT scan was performed using an 0.8 s rotation time, 80 mA, 140 kVp, and a 5 mm slice thickness in high-speed mode with the patient's arms raised over their head. Whole-body ^18^F-FDG PET/CT scan was acquired in the 2-dimensional acquisition mode with 3 min/bed position. After data acquisition, attenuation correction of the PET emission data was performed by CT-based attenuation correction (CTAC). Image reconstruction was performed with an ordered-subset expectation maximization (OSEM) iterative algorithm (2 iterations, 28 subsets).

The acquired PET and CT images were sent to an Xeleris (GE Medical Systems) workstation for registration and fusion. The PET image, CT image, and fused PET/CT image were reviewed by 2 experienced physicians each with more than 10 years of experience in nuclear medicine. For qualitative analysis, the degree of FDG activity in the nodules was defined as either negative (i.e., less than mediastinal blood-pool activity) or positive (i.e., same as or greater than mediastinal blood-pool activity). For semiquantitative analysis, the region of interest (ROI) was drawn along the margin of the lesion for the measurement of the maximum standardized uptake value (SUVmax). In patients with negative PET/CT images, ROIs were drawn on the chest CT and copied to same region on the PET/CT image. SUVmax was measured to represent the ^18^F-FDG uptake of the lesion.

### 2.3. Thin-Section CT Examination

Thin-section CT of the nodules was performed using the GE Discovery LS PET/CT scanner with 140 kVp, 160 mA, and a pitch of 0.875. Thin-section CT images were reconstructed into 1.0 mm-thick sections using high-frequency algorithms.

The thin-section CT images were displayed with lung (level, −600 HU; width, 1700 HU) and mediastinal (level, 30 HU; width, 400 HU) window settings in a multiplanar format and were reviewed separately by two experienced chest radiologists. On CT images, nodules were classified into GGO, solid and mixed nodules. GGO was defined as focal nodular areas of hazy increased lung attenuation with preservation of bronchial and vascular margins. Solid nodule was defined as an opacity with the density similar to that of soft tissue. The mixed nodule was considered when GGO and solid component were mixed in an opacity. The percentage of GGO was calculated as [(*D*_GGO_ − *D*)/*D*_GGO_] × 100, where *D*_GGO_ is the greatest diameter of the lesion, including the GGO area and *D* is the greatest diameter of the lesion without GGO [12].

### 2.4. Statistical Analysis

The descriptive data were expressed as mean ± standard deviation. One-way ANOVA was used to analyze continuous variables, and the Pearson Chi-Square test was used to compare categorical variables between groups. The SUVmax was correlated with GGO percentage with linear regression analysis. General linear model factor analysis was used to analyze the influence of GGO and lesion size on false-negative PET/CT results. A *P* value of 0.05 or less was considered significant. SPSS 13.0 software (SPSS, Inc., Chicago, IL) was used for all analyses.

## 3. Results

The ^18^F-FDG PET/CT results were positive in 58.8% (30/51) of patients with adenocarcinoma with BAC features, and, in 21 patients, the ^18^F-FDG PET/CT results were negative. The SUVmax of the PET/CT positive BAC group was significantly higher than that of the PET/CT negative BAC group (7.79 ± 4.08 versus 1.29 ± 0.63, resp.; *t* = 7.20, *P* = 0.000).

**Figure 1:**
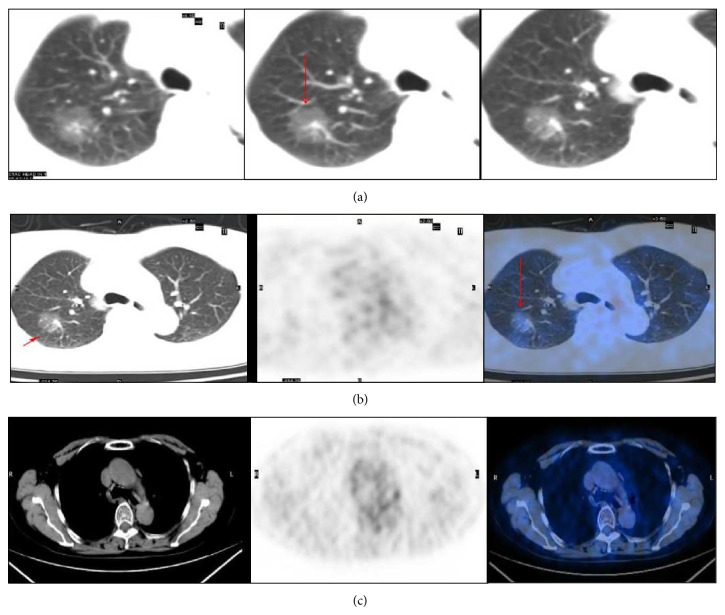
A 75-year-old female with an adenocarcinoma lesion with BAC features (red arrow). (a) Axial thin section CT images revealed a 2.8 cm pure ground-glass opacity in the right upper lobe. (b, c) ^18^F-FDG PET/CT was negative for the lesion, and the maximum standardized uptake value = 0.8.

**Figure 2:**
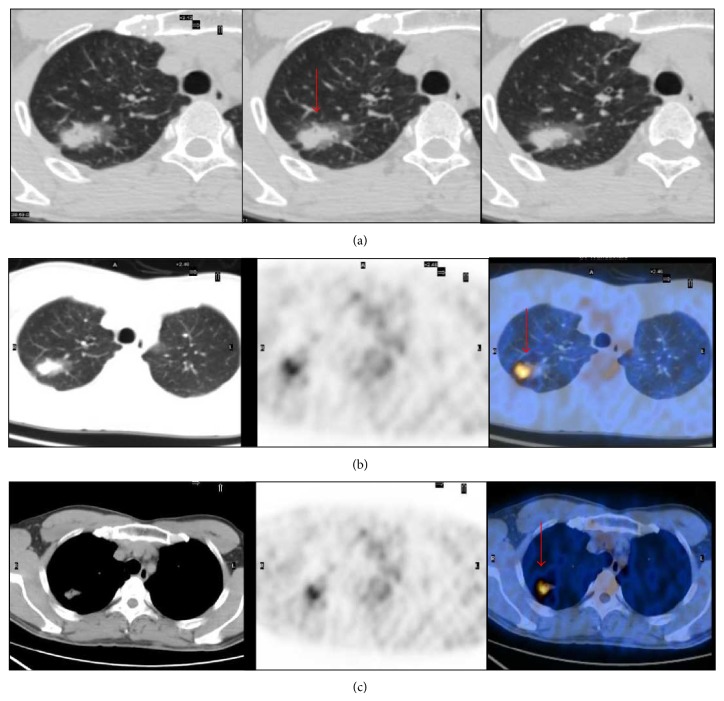
A 47-year-old male with an adenocarcinoma lesion with BAC features (red arrow). (a) Axial thin section CT image revealed a 2.3 cm mixed ground-glass opacity nodule in right upper lobe. (b, c) ^18^F-FDG PET/CT was positive for the lesion, and the maximum standardized uptake value = 3.59. Focal hypermetabolism was localized in the soft part of the lesion but not in the ground-glass part.

**Figure 3:**
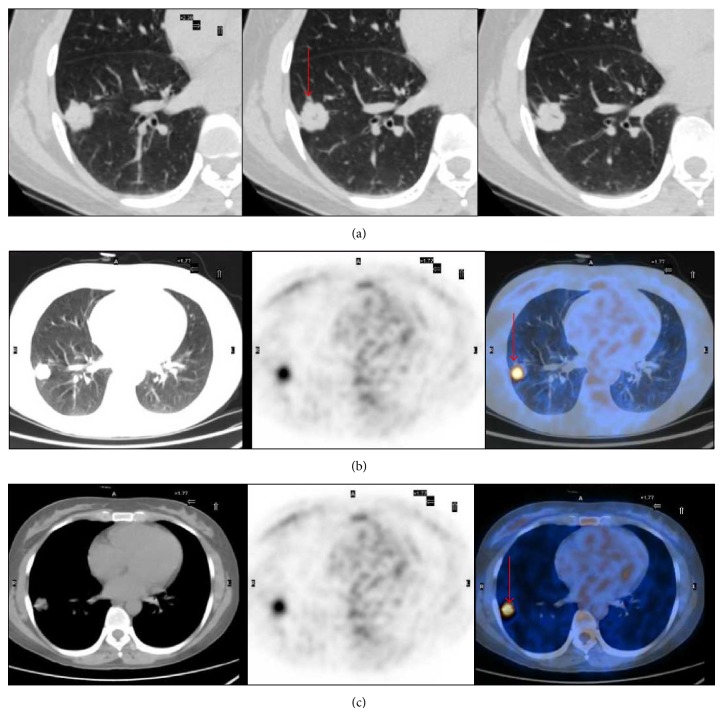
A 47-year-old woman with an adenocarcinoma lesion with BAC features (red arrow). (a) Axial thin section CT image revealed a 2.2 cm solid nodule (arrow) in right lower lobe. (b, c) ^18^F-FDG PET/CT was positive for the lesion, and the maximum standardized uptake value = 5.66.

Of the 51 cases, 19.6% of patients presented with GGO nodules, 31.4% with mixed nodules, and 49.0% with solid nodules. The different types of lesions exhibited different ^18^F-FDG uptake on PET/CT images. ^18^F-FDG PET/CT demonstrated positive detection in 0.0% of the pure GGO nodules, 37.5% of the mixed nodules, and 96.0% of solid nodules (Table 1). Examples of the different types of lesions with different ^18^F-FDG uptake patterns were shown in Figures 1–3. In the mixed nodule group, the SUVmax of the nodules was negatively correlated with GGO percentage (mean, 68%; range, 54–93%) (*r* = −0.588, *P* = 0.021). Similar to the visual analysis, ^18^F-FDG uptake in the lesions of the 3 groups were significantly different (*F* = 20.827, *P* = 0.000, Table 1). The SUVmax of GGO nodule and mixed nodule groups was significantly lower than that of the solid nodules group (*P* = 0.000). No significant difference between the SUVmax of the GGO nodules group and mixed nodules group was observed (*P* = 0.170).

Twenty patients had tumors from 1.1 to 2.0 cm in diameter, 18 from 2.1 to 3.0 cm in diameter, and 7 > 3.0 cm in diameter. Analysis showed that SUVmax was significantly positively correlated with tumor size (*r* = 0.60, *P* < 0.01). Lower ^18^F-FDG uptake in lesions 1.2–2.0 cm in diameter was noted as compared with lesions 2.1–3.0 cm and lesions > 3.0 cm in diameter (both, *P* = 0.000). However, ^18^F-FDG PET/CT exhibited no significant difference in the positive detection rate among the 3 tumor size groups (*χ* ^2^ = 5.815, *P* = 0.055, Table 1). Approximately the same positive detection rate was found in the 1.1–2.0 cm and 2.1–3.0 cm diameter groups (50.0% versus 55.6%, resp.; *P* > 0.05), but all lesions > 3.0 cm in diameter were ^18^F-FDG avid (Table 1).

Factorial design ANOVA was used to analyze the influence of GGO and lesion size on false negativity of PET/CT results. Analysis demonstrated that GGO had a statistically significant effect on false-negative PET/CT results of BAC lesions (*F* = 23.992, *P* = 0.000); however, lesion size had no effect (*F* = 0.602, *P* = 0.866). The results also showed that there was no interaction between GGO and lesion size with respect to false-negative PET/CT results (*F* = 1.069, *P* = 0.446).

## 4. Discussion

Although ^18^F-FDG PET/CT is a valuable imaging modality for the diagnosis and staging of lung cancer, it has several pitfalls. Focal adenocarcinoma with BAC features has been reported as often being negative on ^18^F-FDG PET/CT scans [7, 8, 13, 14]. Sun et al. [15] reported that the SUVmax of adenocarcinoma with BAC (mean, 7.2) was significantly lower than that of other subtypes of NSCLC (mean, 13.33) (*P* < 0.0001). A low sensitivity (72%) of ^18^F-FDG PET/CT for detecting adenocarcinoma with BAC features was reported by Khandani et al. [16] Heyneman and Patz Jr. [17] also reported that PET/CT failed to identify 40% of adenocarcinoma lesions with BAC features. In the present study, ^18^F-FDG PET/CT showed a similar low sensitivity (58.8%) for diagnosing adenocarcinoma with BAC features.

Because of the low positive detection rate, ^18^F-FDG PET/CT seems to be unsuitable for imaging adenocarcinoma with BAC features. However, the results of the present study indicated that about 50% of adenocarcinoma with BAC features were PET/CT positive, and patients with adenocarcinoma with BAC features that are PET/CT positive may also potentially benefit from PET/CT staging or postoperative surveillance. However, the problem lies in predicting which type of adenocarcinoma with BAC features may potentially benefit from PET/CT.

In the present study, none of the BAC lesions with pure GGO was positive on PET/CT. A similar result was reported by Nomori et al. [18] that false-negative PET/CT results were found in 90% of GGO lesions. Very low ^18^F-FDG uptake in pure GGO lesions was also reported by Goudarzi et al. [19]. In their study, a total of 26 pure GGO lesions had a median SUVmax of 1.48 (range, 0.63–4.54), and a SUVmax < 2.5 was found in 81% of the lesions. GGO is commonly seen in adenocarcinoma lesions with BAC features and is thought to be derived from the combined effects of reduction of alveolar air spaces and increased cellular components with alveolar cuboidal cell hyperplasia, thickening of alveolar septa, and partial filling of the alveolar air spaces by tumor cells [7, 20]. Lower ^18^F-FDG uptake in GGO lesions may be the result of the low metabolic demand of slow-growing lesions or a very small number of active malignant cells in these lesions.

Being contrast to the pure GGO lesions, the present study demonstrated that nearly all of the solid nodules were positive of ^18^F-FDG PET. It is similar to the results reported by Lee et al. [21]. Their results showed that the mean SUVmax 2.3 ± 1.9 for mucinous BACs, which appear as solid or part-solid nodules on CT, was significantly higher than that of 0.5 ± 0.8 for nonmucinous BACs, which present as pure GGO nodules (*P* = 0.007). The solid nodules may be predominantly composed of actively growing malignant cells and thus a high glucose demand. Because pure GGO nodule and solid nodule showed a nearly completely different ^18^F-FDG uptake pattern, we suggested that ^18^F-FDG PET/CT might be suitable for imaging of adenocarcinoma lesions with BAC features which present as solid nodule on CT and should not be used for imaging of those as GGO nodule.

The present study also suggests that it is the proportion of the solid component in the adenocarcinoma lesions with BAC features which actually determines whether the tumor is positive or not on PET/CT. Liu et al. found that the percentage of GGO was negatively correlated with the ^18^F-FDG uptake in the lesion [22]. The present study demonstrated a similar tendency in the mixed nodule group (*r* = −0.588; *P* = 0.021). As shown in Figure 2, focal hypermetabolism was localized in the soft part of the lesion but not in the ground-glass part. Mixed nodules may represent an intermediate stage of tumor growth, that is, from the indolent stage of a pure GGO lesion to active tumor. Because of the low positive detection rate, we suggested that ^18^F-FDG PET/CT might not be suitable for imaging of adenocarcinoma lesions with BAC features which present as mixed nodules on CT.

Small NSCLC lesions are often found to have low ^18^F-FDG uptake and are prone to be false-negative on PET/CT [10, 23]. Although the present study also demonstrated the SUVmax of BAC lesions and tumor size had a significant positive correlation (*r* = 0.657, *P* = 0.000), the factorial design ANOVA study indicated the tumor size was not associated with false-negative PET/CT results. Approximately the same positive detection rate of ^18^F-FDG PET/CT was found for lesions 1.1–2.0 cm and lesions 2.1–3.0 cm in diameter (50% versus 55.6%, resp.; *P* > 0.05). Factorial design ANOVA demonstrated that GGO had a statistically significant effect on false-negative PET/CT results of adenocarcinoma lesions with BAC features but not lesion size. This result was different from what was found in the adenocarcinoma lesions without BAC features. In the adenocarcinoma lesions without BAC features, the lesion size was often significantly associated with ^18^F-FDG uptake [24]. This difference may result from the different presentations between them. Most adenocarcinoma lesions without BAC features are solid lesions; however, ground-glass changes are commonly seen in adenocarcinoma lesions with BAC features.

A limitation of the present study is the small sample size of the mixed nodule group, and all of the lesions were ≥ 1.0 cm. Further studies with a larger patient group, including patients with adenocarcinoma lesions with BAC features < 1.0 cm are needed. In addition, the retrospective nature of the study may limit the interpretation of the results.

## 5. Conclusion

Adenocarcinoma with BAC features is considered as the main cause of falsely negative finding on ^18^F-FDG PET/CT. However, the present study demonstrated that different types of adenocarcinoma with BAC features exhibited different ^18^F-FDG uptake patterns. False-negative on ^18^F-FDG PET/CT mainly occurs in those lesions which presented as the nonsolid nodule on CT but not the solid nodule. A patient diagnosed with adenocarcinoma lesions with BAC features may still benefit from postoperative ^18^F-FDG PET/CT surveillance when the lesion presented as a solid nodule on the preoperative CT images. Further research is needed to confirm the present results.

## Figures and Tables

**Table 1:** GGO component, lesion size, SUVmax, and PET/CT positivity in adenocarcinoma lesions with BAC features.

Tumor	Number	PET/CT positivity, no. (%) of nodules	SUVmax
GGO component		*χ* ^2^ = 31.55, *P* = 0.000	*F* = 20.827, *P* = 0.000
GGO nodule	10	0 (0.0)	1.04 ± 0.43
Mixed nodule	16	6 (37.5)	2.93 ± 2.99
Solid nodule	25	24 (96.0)	8.14 ± 4.11
Lesion size (cm)		*χ* ^2^ = 5.815, *P* = 0.055	*F* = 21.463, *P* = 0.000
1.1–2.0	26	13 (50.0)	2.20 ± 2.51
2.1–3.0	18	10 (55.6)	7.46 ± 3.59
>3.0	7	7 (100)	9.88 ± 5.10

BAC, bronchoalveolar cell carcinoma; GGO, ground-glass opacity; SUVmax, maximum standardized uptake value.

## References

[1] Travis W. D., Garg K., Franklin W. A. (2005). Evolving concepts in the pathology and computed tomography imaging of lung adenocarcinoma and bronchioloalveolar carcinoma. *Journal of Clinical Oncology*.

[2] Read W. L., Page N. C., Tierney R. M., Piccirillo J. F., Govindan R. (2004). The epidemiology of bronchioloalveolar carcinoma over the past two decades: analysis of the SEER database. *Lung Cancer*.

[3] Arenberg D. (2011). Bronchioloalveolar carcinoma. *Seminars in Respiratory and Critical Care Medicine*.

[4] Gaeta M., Blandino A., Pergolizzi S. (2003). Patterns of recurrence of bronchioloalveolar cell carcinoma after surgical resection: a radiological, histological, and immunohistochemical study. *Lung Cancer*.

[5] Lee H. Y., Lee K. S., Han J. (2009). Mucinous versus nonmucinous solitary pulmonary nodular bronchioloalveolar carcinoma: CT and FDG PET findings and pathologic comparisons. *Lung Cancer*.

[6] AL-Jahdali H., Khan A. N., Loutfi S., Al-Harbi A. S. (2012). Guidelines for the role of FDG-PET/CT in lung cancer management. *Journal of Infection and Public Health*.

[7] Ambrosini V., Nicolini S., Caroli P. (2012). PET/CT imaging in different types of lung cancer: an overview. *European Journal of Radiology*.

[8] Lee H. Y., Lee K. S. (2011). Ground-glass opacity nodules: histopathology, imaging evaluation, and clinical implications. *Journal of Thoracic Imaging*.

[9] Huang T.-W., Lin L.-F., Hsieh C.-M. (2012). Positron emission tomography in bronchioloalveolar carcinoma of the lung. *European Journal of Surgical Oncology*.

[10] Suzawa N., Ito M., Qiao S. (2011). Assessment of factors influencing FDG uptake in non-small cell lung cancer on PET/CT by investigating histological differences in expression of glucose transporters 1 and 3 and tumour size. *Lung Cancer*.

[11] Delbeke D., Coleman R. E., Guiberteau M. J. (2006). Procedure guideline for tumor imaging with ^18^F-FDG PET/CT 1.0. *Journal of Nuclear Medicine*.

[12] Kim T. J., Park C. M., Goo J. M., Lee K. W. (2012). Is there a role for FDG PET in the management of lung cancer manifesting predominantly as ground-glass opacity?. *The American Journal of Roentgenology*.

[13] Erasmus J. J., MacApinlac H. A. (2012). Low-sensitivity FDG-PET studies: less common lung neoplasms. *Seminars in Nuclear Medicine*.

[14] Aquino S. L., Halpern E. F., Kuester L. B., Fischman A. J. (2007). FDG-PET and CT features of non-small cell lung cancer based on tumor type. *International Journal of Molecular Medicine*.

[15] Sun J. S., Park K. J., Sheen S. S. (2009). Clinical usefulness of the fluorodeoxyglucose (FDG)-PET maximal standardized uptake value (SUV) in combination with CT features for the differentiation of adenocarcinoma with a bronchioloalveolar carcinoma from other subtypes of non-small cell lung cancers. *Lung Cancer*.

[16] Khandani A. H., Whitney K. D., Keller S. M., Isasi C. R., Donald Blaufox M. (2007). Sensitivity of FDG PET, GLUT1 expression and proliferative index in bronchioloalveolar lung cancer. *Nuclear Medicine Communications*.

[17] Heyneman L. E., Patz E. F. (2002). PET imaging in patients with bronchioloalveolar cell carcinoma. *Lung Cancer*.

[18] Nomori H., Watanabe K., Ohtsuka T., Naruke T., Suemasu K., Uno K. (2004). Evaluation of F-18 fluorodeoxyglucose (FDG) PET scanning for pulmonary nodules less than 3 cm in diameter, with special reference to the CT images. *Lung Cancer*.

[19] Goudarzi B., Jacene H. A., Wahl R. L. (2008). Diagnosis and differentiation of bronchioloalveolar carcinoma from adenocarcinoma with bronchioloalveolar components with metabolic and anatomic characteristics using PET/CT. *Journal of Nuclear Medicine*.

[20] Kushihashi T., Munechika H., Ri K. (1994). Bronchioloalveolar adenoma of the lung: CT-pathologic correlation. *Radiology*.

[21] Lee H. Y., Han J., Lee K. S. (2009). Lung adenocarcinoma as a solitary pulmonary nodule: prognostic determinants of CT, PET, and histopathologic findings. *Lung Cancer*.

[22] Liu S., Cheng H., Yao S. (2010). The clinical application value of PET/CT in adenocarcinoma with bronchioloalveolar carcinoma features. *Annals of Nuclear Medicine*.

[23] Tsunezuka Y., Shimizu Y., Tanaka N., Takayanagi T., Kawano M. (2007). Positron emission tomography in relation to Noguchi's classification for diagnosis of peripheral non-small-cell lung cancer 2 cm or less in size. *World Journal of Surgery*.

[24] Iwano S., Ito S., Tsuchiya K., Kato K., Naganawa S. (2013). What causes false-negative PET findings for solid-type lung cancer?. *Lung Cancer*.

